# Microbiome Compositions From Infertile Couples Seeking *In Vitro* Fertilization, Using 16S rRNA Gene Sequencing Methods: Any Correlation to Clinical Outcomes?

**DOI:** 10.3389/fcimb.2021.709372

**Published:** 2021-10-01

**Authors:** Somadina I. Okwelogu, Joseph I. Ikechebelu, Nneka R. Agbakoba, Kingsley C. Anukam

**Affiliations:** ^1^ Department of Medical Laboratory Science, Faculty of Health Sciences & Technology, Nnamdi Azikiwe University, Nnewi, Nigeria; ^2^ Department of Obstetrics and Gynaecology, Nnamdi Azikiwe University Teaching Hospital, Nnewi, Nigeria; ^3^ Department of Pharmaceutical Microbiology, Faculty of Pharmaceutical Sciences, Nnamdi Azikiwe University, Awka, Nigeria; ^4^ Department of Research and Development, Uzobiogene Genomics, London, ON, Canada

**Keywords:** seminal fluid, microbiome, *in vitro* fertilization - pregnancy, infertility, bacteria infections, vagina, 16S rRNA, sequencing

## Abstract

**Background:**

Bacterial infections are usually suspected in infertile couples seeking IVF with no clear understanding of the microbial compositions present in the seminal fluids and vaginal niche of the patients. We used next-generation sequencing technology to correlate microbiota compositions with IVF clinical outcomes.

**Methods:**

Thirty-six couples were recruited to provide seminal fluids and vaginal swabs. Bacterial DNA was extracted, and V4 region of the 16S rRNA was amplified and sequenced in a pair-end configuration on the Illumina MiSeq platform rendering 2 × 150 bp sequences. Microbial taxonomy to species level was generated using the Greengenes database. Linear discriminant analysis (LDA) effect size (LEfSe) was used to identify biologically and statistically significant differences in relative abundance.

**Results:**

Seminal fluid microbiota compositions had lower bacterial concentrations compared with the vagina, but species diversity was significantly higher in seminal fluid samples. Azoospermic subjects had more relative abundance of *Mycoplasma* and *Ureaplasma.* In Normospermic semen, *Lactobacillus* (43.86%) was the most abundant, followed by *Gardnerella* (25.45%), while the corresponding vaginal samples, *Lactobacillus* (61.74%) was the most abundant, followed by *Prevotella* (6.07%) and *Gardnerella* (5.86%).

**Conclusions:**

Semen samples with positive IVF were significantly colonized by *Lactobacillus jensenii* (*P*=0.002), *Faecalibacterium* (*P*=0.042) and significantly less colonized by *Proteobacteria*, *Prevotella*, *Bacteroides*, and lower *Firmicutes*/*Bacteroidetes* ratio compared with semen samples with negative IVF. Vaginal samples with positive IVF clinical outcome were significantly colonized by *Lactobacillus gasseri*, less colonized by *Bacteroides* and *Lactobacillus iners.* This study has opened a window of possibility for *Lactobacillus* replenishments in men and women before IVF treatment.

## Introduction

Bacterial infections affecting the reproductive tracts of males and/or females with infertility have been documented in previous studies using culture methods (Eggert-Kruse et al., 1995; Gdoura et al., 2008). Some bacteria, fungi, viruses, and parasites are known to interfere with reproductive functions in both male and females of reproductive age, and infections of the genitourinary tract account for about 15% of male infertility cases. More than a few bacteria, including *Lactobacillus iners*, *Gardnerella vaginalis*, *Escherichia faecalis*, *E. coli*, and *Staphylococcus aureus*, have been found to be associated with male infertility as demonstrated using polymerase chain reaction (PCR) (Franasiak and Scott, 2015). Bacterial vaginosis (BV) has been found to be a major risk factor for infertility (Al-Awadhi et al., 2013). Some specific bacteria incriminated in BV, such as *Atopobium vaginae*, *Ureaplasma vaginae*, *U. parvum*, *U. urealyticum*, *Gardnerella vaginalis*, and reduced abundance of *Lactobacillus* species, are associated with infertility in women (Onemu and Ibeh, 2001). Women with endometrial dysbiosis have also been found to experience implantation failure leading to infertility (Momoh et al., 2007). While vaginal microbiota is normally under the influence of oestrogen (Ibadin and Kai, 2008), endometrial microbiota is not affected by hormonal fluctuations (Ekhaise and Richard, 2001).

There are several studies confirming that a vaginal microbiota replete with relative abundances of *Lactobacillus* species, devoid of bacterial vaginosis, leads to more positive IVF clinical outcomes (Nwadioha et al., 2016).

Besides other sexually transmitted infections, genital mycoplasmas are associated with poor reproductive health of women including but not limited to endometritis, cervicitis and pelvic inflammatory disease, and adverse pregnancy outcomes (Lis et al., 2015; Taylor et al., 2018). The pregnancy success rate of the various assisted reproductive health care such as *in vitro* fertilization (IVF) tend to be reduced as a result of prior *Mycoplasma* colonization of the female and male genital tract (Günyeli et al., 2011). One wonders whether genital *Mycoplasma* was the only pathogen associated with poor pregnancy outcome, although several studies have shown that *Mycoplasma* species can attach to spermatozoa and remain adherent to spermatozoa after assisted reproductive treatment washing procedures (Ibadin and Osemwenkha, 2013; Ahmadi et al., 2018).

Clinical studies have shown that bacterial contamination of the embryo transfer catheter has significant negative effect on the clinical pregnancy rates (Ikechebelu, 2003 **)**. Approximately 35% of infertile women are afflicted with post-inflammatory changes of the oviduct or surrounding peritoneum that interfere with tubal-ovarian function mostly as a result of infection and are likely to develop ectopic (tubal) pregnancy (Swift and Liu, 2014).

In Africa, especially Nigeria, little is known about the bacterial communities found in the seminal fluids of men seeking reproductive health care with next-generation sequencing technology. A recent pilot study of seminal fluid in a tertiary hospital revealed varying bacterial community diversities that are unique in each sample in contrast to culture-dependent methods (Ndiokwere et al., 2019). We have also previously shown that women with bacterial vaginosis (BV) had varying proportions of diverse bacteria including *Lactobacillus* species in all BV subjects, but the total number of all the BV-associated microbes (*Gardnerella*, *Prevotella*, *Magasphaera*, and others) outnumbered *Lactobacillus* genera (Anukam et al., 2019). In the present study, next-generation 16S rRNA gene sequencing method was used to compare seminal bacterial composition in couples seeking reproductive health care IVF. In addition, the study delineated semen quality, bacterial functional gene predictions, and correlated microbiota composition with clinical outcome of the IVF-assisted reproductive care.

## Materials and Methods

### Ethical Approval

The study was approved by the Ethics Review Committee on Human Research from Nnamdi Azikiwe University Teaching Hospital (Ref # NAUTH/CS/66/VOL11/175/2018/111).

Participation in the study was voluntary. Informed written consent was obtained from the patients. All methods were performed in accordance with the relevant guidelines and regulations.

### Selection Criteria

Couples seeking reproductive health care at Nnamdi Azikiwe University Teaching Hospital (NAUTH), Nnewi campus, Anambra State, Nigeria, were referred to Life Fertility Center, Nnewi, for IVF/Embryo Transfer for self-cycle with cases of primary or secondary infertility after 1–12 years of uninterrupted sexual intercourse with partner.

### Collection of Specimens

Two high vaginal swabs were collected by a qualified gynecologist with a non-lubricated sterile disposable plastic speculum. One of the swabs was agitated into a tube containing buffer for DNA preservation at ambient temperature, and the other was used for microscopy to detect leukocytes. Each semen sample was produced by masturbation after 5 days of sexual intercourse abstinence, and on the same day vaginal sample was collected.

### Semen Analysis

The semen quality of the patients was analyzed with Semen Quality Analyzer-Vision (SQA-V) Gold (Medical Electronic Systems, USA), following the manufacturer’s procedural instructions.

### Extraction of Bacterial DNA From Vaginal Swabs/Semen Samples and Sequencing of the Amplified 16S rRNA Region

Bacterial DNA was extracted from the vaginal swabs/semen samples using a protocol developed by uBiome Inc. Briefly, samples were lysed using bead-beating, and DNA was extracted in a class 1,000 clean room by a guanidine thiocyanate silica column-based purification method using a liquid-handling robot. PCR amplification of the 16S rRNA genes was performed with primers containing universal primers amplifying the V4 region (515F: GTGCCAGCMGCCGCGGTAA and 806R: GGACTACHVGGGTWTCTAAT). In addition, the primers contained Illumina tags and barcodes. Samples were barcoded with a unique combination of forward and reverse indexes allowing for simultaneous processing of multiple samples. PCR products were pooled, column-purified, and size-selected through microfluidic DNA fractionation. Consolidated libraries were quantified by quantitative real-time PCR using the Kapa Bio-Rad iCycler qPCR kit on a BioRad MyiQ before loading into the sequencer. Sequencing was performed in a pair-end modality on the Illumina NextSeq 500 platform rendering 2 × 150 bp pair-end sequences (Almonacid et al., 2017).

### 16S rRNA Sequence Analysis

Raw sequence reads were demultiplexed using Illumina’s BCL2FASTQ algorithm. Reads were filtered using an average Q-score >30. The paired-end sequence FASTQ reads were imported into MG-RAST pipeline for quality check (QC). EzBiocloud Microbiome Taxonomic Profile (MTP) pipeline (Yoon et al., 2017) was employed for alpha and beta diversity estimation using PKSSU4.0 version database and Open reference UCLUST_MC2 for OTUs picking at 97% cutoff. Sequences were prescreened using QIIME-UCLUST algorithms for at least 97% identity to ribosomal sequences from the RNA databases (Quast et al., 2013). Rarefication to 1,000 reads per sample was employed to calculate microbial diversity. Alpha diversity was calculated for species richness by Abundance Coverage Estimate (ACE), Chao1 and Jackknife method, while diversity indexes were calculated by Shannon, Non-parametric Shannon, and Simpson index. Principal coordinate analysis (PCoA) with Jensen-Shannon divergence distance metrices were used to evaluate beta diversity between vaginal and semen samples (Koren et al., 2013). Linear discriminant analysis (LDA) effect size (LEfSe) (Segata et al., 2011) was used to identify biologically and statistically significant differences in the OTU relative abundance. Phylogenetics Investigation of Communities by Reconstruction of Unobserved States (PICRUSt) was used to predict the metabolic function of the metagenomes from the 16S rRNA gene dataset (Langille et al., 2013) with reference to Kyoto Encyclopedia of Genes and Genomes (KEGG) Orthologs categorizations (Kanehisa et al., 2014).

### Availability of Data and Materials

The datasets used and or analyzed in the current study are available from the corresponding author on reasonable request.

## Results

### Demographic Information, IVF Clinical Outcome, and Semen Quality

As shown in **Table 1A** **/** **Table 1B**, of the 36 men that were examined for semen quality and that had result for 16S rRNA gene sequencing, 11 were clinically diagnosed as having secondary infertility with duration of infertility ranging from 1 to 13 years, while 25 men were diagnosed with primary infertility, had duration of infertility from 1 to 8 years. The semen characteristics of the subjects were as follows: 11 samples were assigned as normospermia [>15×10 (6)], 7 had oligospermia [<15×10 (6)], 7 had azoospermia, 10 had asthenozoospermia, while 1 sample was classified as tetratozoospermia. The number of semen samples that had leukocytes (pyospermia) were 11, while 1 sample was assigned as oligoasthenozoospermia.

**Table 1A:** Demographic information and IVF clinical outcome.

Sample No.	Female age (years)	Clinical pregnancy outcome	Scan result	Sex of the baby	Live delivery	Male age (years)	Duration of marriage (years)	Duration of infertility (years)	Type of infertility
1	26–30	Negative	–	**-**		31–35	6	6	Primary
2	36–40	Negative	–	**-**		41–45	10	8	Secondary
3	36–40	Negative	–	**-**		46–50	6	6	Primary
4	31–35	Negative	–	**-**		36–40	10	10	Primary
5	26–30	Negative	–	**-**		36–40	5	5	Primary
6	26–30	Negative	–	**-**		26–30	4	4	Primary
7	36–40	POSITIVE	Singleton	Male	YES	46–50	9	8	Secondary
8	31–35	Negative	–	–		31–35	3	3	Primary
9	21–25	Negative	–	–		26–30	4	4	Primary
10	36–40	POSITIVE	Singleton	Female	YES	36–40	5	5	Primary
11	41–45	POSITIVE	Twins	Male, Female	YES	46–50	3	2	Secondary
12	36–40	Negative	–	–		41–45	4	4	Primary
13	26–30	Negative	–	–		26–30	1	1	Primary
14	31–35	POSITIVE	Singleton	Male	YES	36–40	2	2	Primary
15	26–30	Negative	–	–		26–30	5	3	Secondary
16	26–30	POSITIVE	Singleton	Male	YES	31–35	2	2	Primary
17	26–30	POSITIVE	Singleton	Male	YES	31–35	3	3	Primary
18	41–45	Negative	–	–		46–50	7	6	Secondary
19	31–35	POSITIVE	Twins	Male, Female	YES	41–45	2	2	Primary
20	26–30	Negative	–	–		31–35	6	6	Primary
21	36–40	Negative	–	–		46–50	9	9	Secondary
22	31–35	Negative	–	–		41–45	4	1	Secondary
23	36–40	POSITIVE	Twins	Male, Female	YES	46–50	12	6	Secondary
24	31–36	Negative	–	**-**		31–35	5	5	Primary
25	26–30	POSITIVE	Singleton	Female	No (Misca)	31–35	3	3	Primary
26	41–45	Negative	–	–		51–55	2	2	Primary
27	31–35	POSITIVE	Singleton	Male	YES	36–40	6	6	Primary
28	31–35	Negative	–	–		31–35	2	2	Primary
29	31–35	Negative	–	–		31–35	4	4	Primary
30	31–35	Negative	–	–		36–40	5	4	Secondary
31	46–50	Negative	–	–		56–60	13	13	Primary
32	36–40	Negative	–	–		46–50	6	6	Primary
33	41–45	POSITIVE	Singleton	Male	YES	51–55	5	5	Primary
34	36–40	Negative	–	–		51–55	10	7	Secondary
35	31–35	POSITIVE	Singleton	Female	YES	41–45	4	1	Secondary
36	36–40	Negative	–	**-**		36–40	6	6	Primary

**Table 1B:** Semen Characteristics.

Sample #	Days of abstinence	Viscosity	Liquefaction	Volume (ml)	Progressive motility (%)	Non-progressive motility (%)	Non-motile (%)	Total motility (%)	Velocity (mic/s)	Sperm conc (m/ml)	Total sperm/volume (million)	Presence of pus cells
1	3	NORMAL	NORMAL	2	9	3	88	12	32	88	176.1	+
2	5	NORMAL	NORMAL	2.5	5	2	93	7	27	22.2	55.4	NIL
3	4	ABNORMAL	ABNORMAL	1.2	0	0	0	0	0	0	0	2+
4	3	NORMAL	NORMAL	2.1	2	9	89	11	0	2	0	NIL
5	3	NORMAL	NORMAL	1.2	27	6	67	33	41	120.5	144.6	NIL
6	4	ABNORMAL	ABNORMAL	1.2	0	0	0	0	0	0	0	2+
7	3	NORMAL	NORMAL	2.8	37	10	53	47	38	77.4	216.7	NIL
8	3	NORMAL	NORMAL	1	44	8	48	52	43	72	72	NIL
9	4	NORMAL	NORMAL	3	22	10	68	32	30	33.8	101.4	NIL
10	5	NORMAL	NORMAL	1.5	39	8	53	47	42	194.5	291.8	NIL
11	3	NORMAL	NORMAL	2.3	51	12	37	63	39	52.7	121.3	NIL
12	5	NORMAL	NORMAL	0.8	17	3	80	20	46	207.6	166.1	NIL
13	4	NORMAL	NORMAL	2	52	20	28	72	32	6.4	12.8	NIL
14	4	ABNORMAL	ABNORMAL	3	0	0	0	0	0	0	0	3+
15	3	NORMAL	NORMAL	3.2	0	1	99	1	0	16	51.1	NIL
16	5	NORMAL	NORMAL	2.2	35	8	57	43	42	77.2	169.8	NIL
17	3	NORMAL	NORMAL	1.5	11	7	82	18	25	66.4	99.7	NIL
18	3	ABNORMAL	ABNORMAL	2.5	0	0	0	0	0	0	0	2+
19	4	NORMAL	NORMAL	1.5	30	6	62	38	43	163.3	244.9	NIL
20	3	ABNORMAL	ABNORMAL	5	0	0	0	0	0	0	0	2+
21	4	NORMAL	NORMAL	3.2	46	9	40	60	44	50.3	160.9	NIL
22	5	ABNORMAL	ABNORMAL	2	0	0	0	0	0	0	0	2+
23	3	NORMAL	NORMAL	2	50	4	84	16	34	157	314	2+
24	3	NORMAL	NORMAL	3.2	47	10	40	60	45	61.6	197.2	NIL
25	3	ABNORMAL	ABNORMAL	2.1	0	0	0	0	0	0	0	2+
26	4	NORMAL	NORMAL	2	5	24	29	29	5	4.7	9.4	NIL
27	3	NORMAL	NORMAL	2.4	3	2	95	5	22	75.3	180	NIL
28	4	NORMAL	NORMAL	2	40	14	46	54	34	44.6	89.2	NIL
29	3	NORMAL	NORMAL	1	3	2	95	5	22	72.2	72.2	NIL
30	5	NORMAL	NORMAL	3	8	19	73	27	10	5.6	16.9	3+
31	3	NORMAL	NORMAL	3.2	1	11	88	12	4	11.3	36	NIL
32	4	ABNORMAL	ABNORMAL	2.1	39	22	39	61	26	5.1	10.8	2+
33	5	NORMAL	NORMAL	1.5	27	6	67	33	40	77.6	116.4	NIL
34	4	NORMAL	NORMAL	1	20	5	75	25	37	134.1	134.1	NIL
35	3	NORMAL	NORMAL	2.5	29	9	62	38	34	80.8	97	NIL
36	5	NORMAL	NORMAL	3	44	7	49	51	45	83.8	251.5	NIL

Key: NIL represents absence of pus cells.

### Semen and Vagina Microbiome Compositions

**Figure 1:**
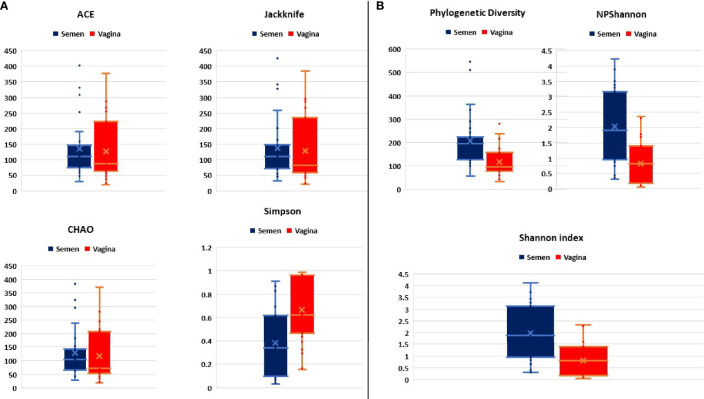
**(A, B)** Comparative alpha diversities between semen and vagina showing species richness typified by ACE, CHAO Jackknife, Shannon, and Simpson index.

The first objective was to determine whether seminal fluid microbiota differ substantially with the vagina. In this regard, the alpha diversity that estimates the species richness typified by ACE, CHAO Jackknife, Shannon, and Simpson shows that seminal fluid microbiota composition is less in species richness (lower bacterial concentrations) compared with the vagina as shown in **Figure 1A**.

**Figure 2:**
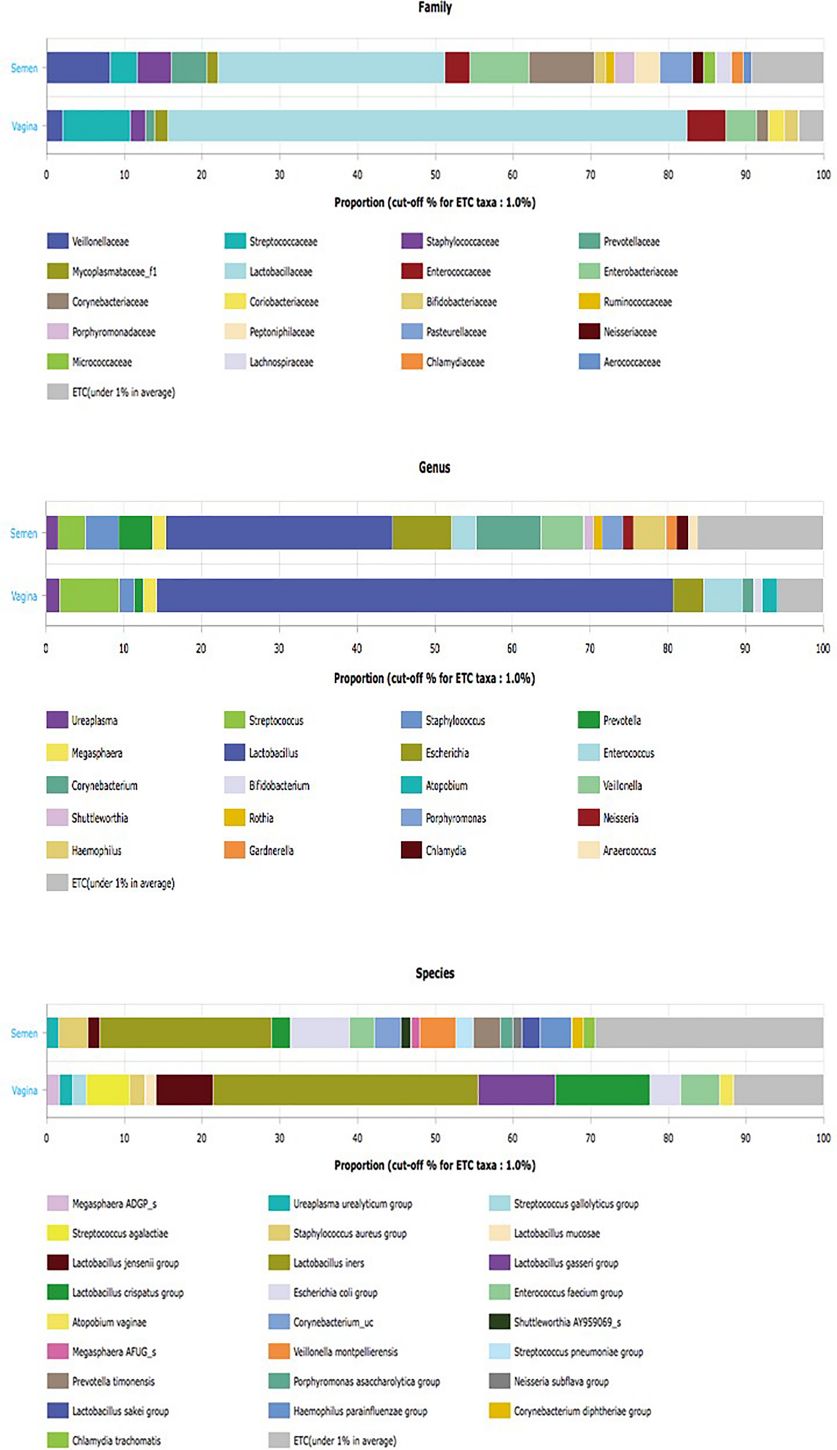
EzBiocloud Microbiome Taxonomic Profile (MTP) pipeline showing distinct taxonomic categories (Family, Genus, and Species) at 1% cutoff between semen and vagina.

However, species diversity was significantly higher in seminal fluid samples represented in **Figure 1B**. The Principal Coordinate Analysis (PCoA) with Bray-Curtis metrices confirmed the differences in bacterial community diversities between semen and vagina as shown in **Supplementary Figure S1**. The EzBiocloud Microbiome Taxonomic Profile (MTP) pipeline was able to provide distinct taxonomic categories (Family, Genus, and Species) at 1% cutoff between semen and vagina as shown in **Figure 2**.

**Figure 3:**
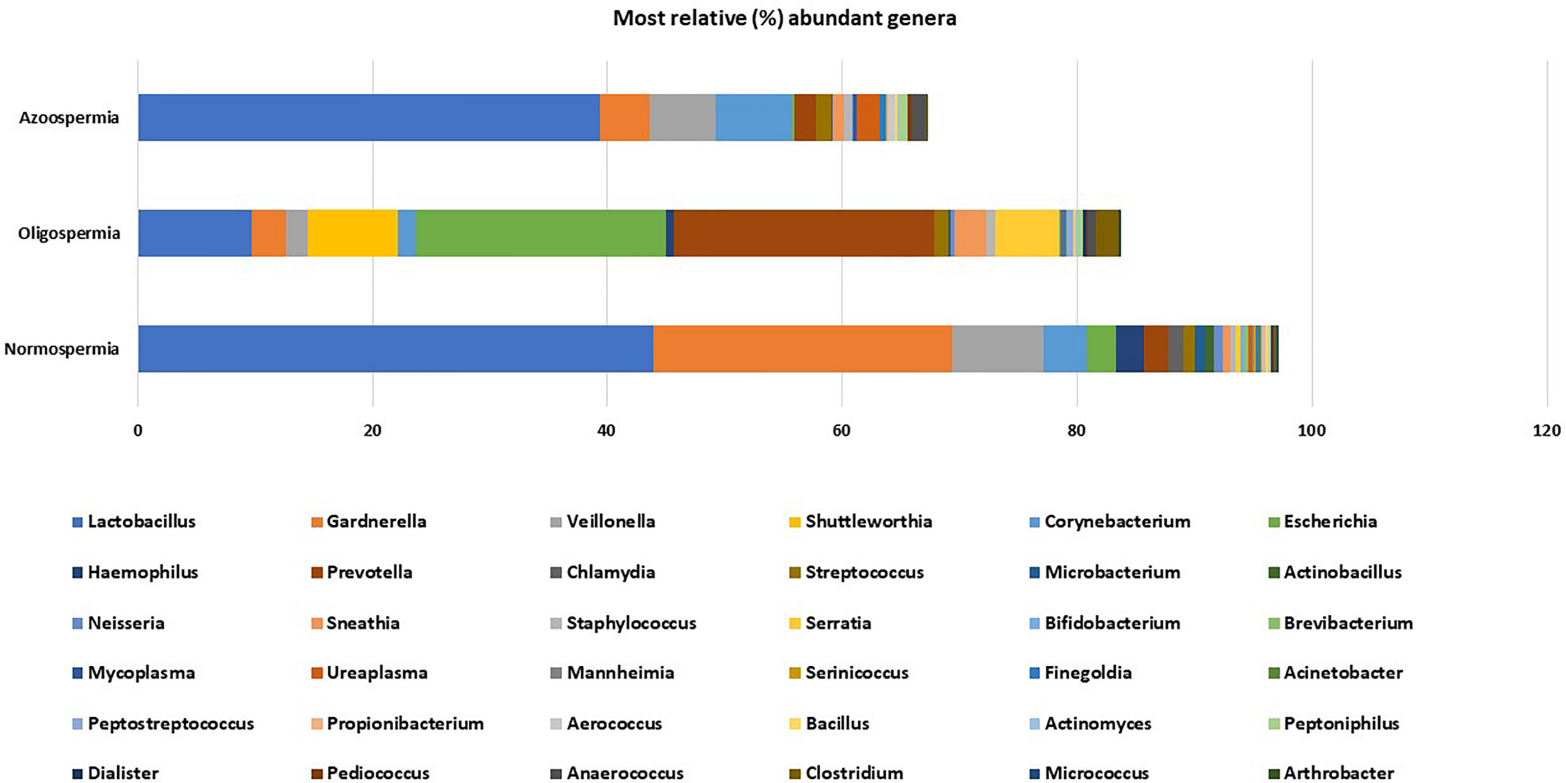
Most relative (%) genera abundance between the semen categories.

The rarefaction curve showing the number of reads between the semen samples and vaginal microbiota is presented in **Supplementary Figure S2**. For comparative purposes, we selected the corresponding semen and vagina bacterial communities in line with the three semen categories. At the genera taxonomic level, out of 621 genera identified, 6.8% were exclusive to normospermia, 20.5% to oligospermia, and 2.9% exclusive to azoospermia, while 41.5% were common to all the categories. When the genera taxa are examined with the three semen categories, *Mycoplasma* and *Ureaplasma* occurred more in relative abundance in azoospermic subjects (**Figure 3**).

At the species taxonomic level, out of 1,384 species identified, 10.3% were exclusive to normospermia, 26.4% to oligospermia, and 6.8% exclusive to azoospermia, while 27% were common to all the categories as shown in **Supplementary Figure S3**.

### Microbiota Compositions From Normospermia Couples

Twenty-four phyla were identified from subjects with normospermia, while 22 were identified from the corresponding vagina samples. *Firmicutes* accounted for 54.47 *vs* 75.89% relative abundance, followed by *Actinobacteria* (32.26 *vs* 8.72%), *Proteobacteria* (8.60 *vs* 7.20%), *Bacteroidetes* (2.29 *vs* 5.95%), *Chlamydiae* (1.32 *vs* 0.0003%), *Fusobacteria* (0.67 *vs* 2.09%), *Tenericutes* (0.30 *vs* 0.09%), and others represented in **Supplementary Figure S4**. At the genera taxonomic level, 451 genera were identified in normospermia samples, while 331 genera were found in the corresponding females. Interestingly, 282 genera were shared between couples. *Lactobacillus* (43.86%) was the most abundant genera in semen/vagina, followed by *Gardnerella* (25.45%), *Veillonella* (7.78%), *Corynebacterium* (3.73%), *Escherichia* (2.47%), *Haemophilus* (2.36%), *Prevotella* (2.03%), and others, while in the corresponding vagina samples, *Lactobacillus* (61.74%) was the most abundant genera, followed by *Prevotella* (6.07%), *Gardnerella* (5.86%), *Streptococcus* (5.84%), *Escherichia* (5.40%), *Megasphaera* (4.51%), *Sneathia* (2.13%), and others represented in **Supplementary Figure S5**.

**Figure 4:**
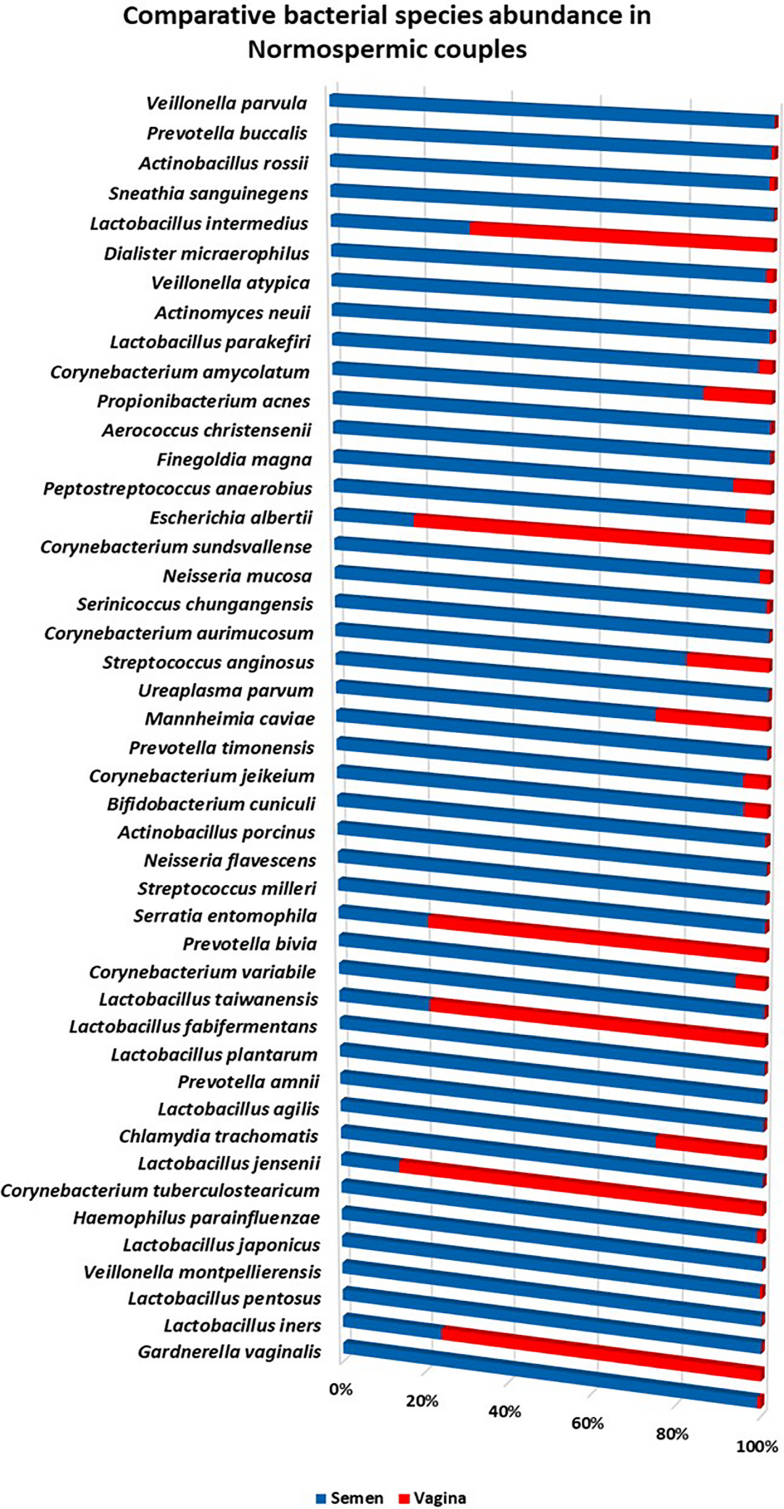
Comparative relative (%) species abundance in normospermia couples.

At the species taxonomic level, 848 species were identified in normospermia samples, while 585 species were found in the corresponding vagina samples. *Gardnerella vaginalis* (31.93%) was the most abundant species identified in 10/12 of the semen samples, followed by *Lactobacillus iners* 8/12 (15.56%), *Lactobacillus pentosus* 1/12 (12.39%), *Veillonella montpellierensis* 8/12 (9.61%), *Lactobacillus japonicus* 3/12 (4.29%), *Haemophilus parainfluenzae* 9/12 (2.91%), *Corynebacterium tuberculostearicum* 11/12 (1.75%), *Lactobacillus jensenii* 3/12 (1.73%), and others. The corresponding vaginal samples had *Lactobacillus iners* 9/12 (49.06%) as the most abundant species, followed by *Lactobacillus jensenii* 2/12 (10.60%), *Peptostreptococcus stomatis* 6/12 (6.92%), *Actinocatenispora silicis* 5/12 (6.19%), *Pasteurella pneumotropica* 2/12 (4.09%), *Actinomyces naturae* 4/12 (3.32%), *Lactobacillus taiwanensis* 10/12 (2.78%), *Peptoniphilus asaccharolyticus* 8/12 (2.69%) and others as shown in **Figure 4**.

### Microbiota Compositions From Oligospermia Couples

**Figure 5:**
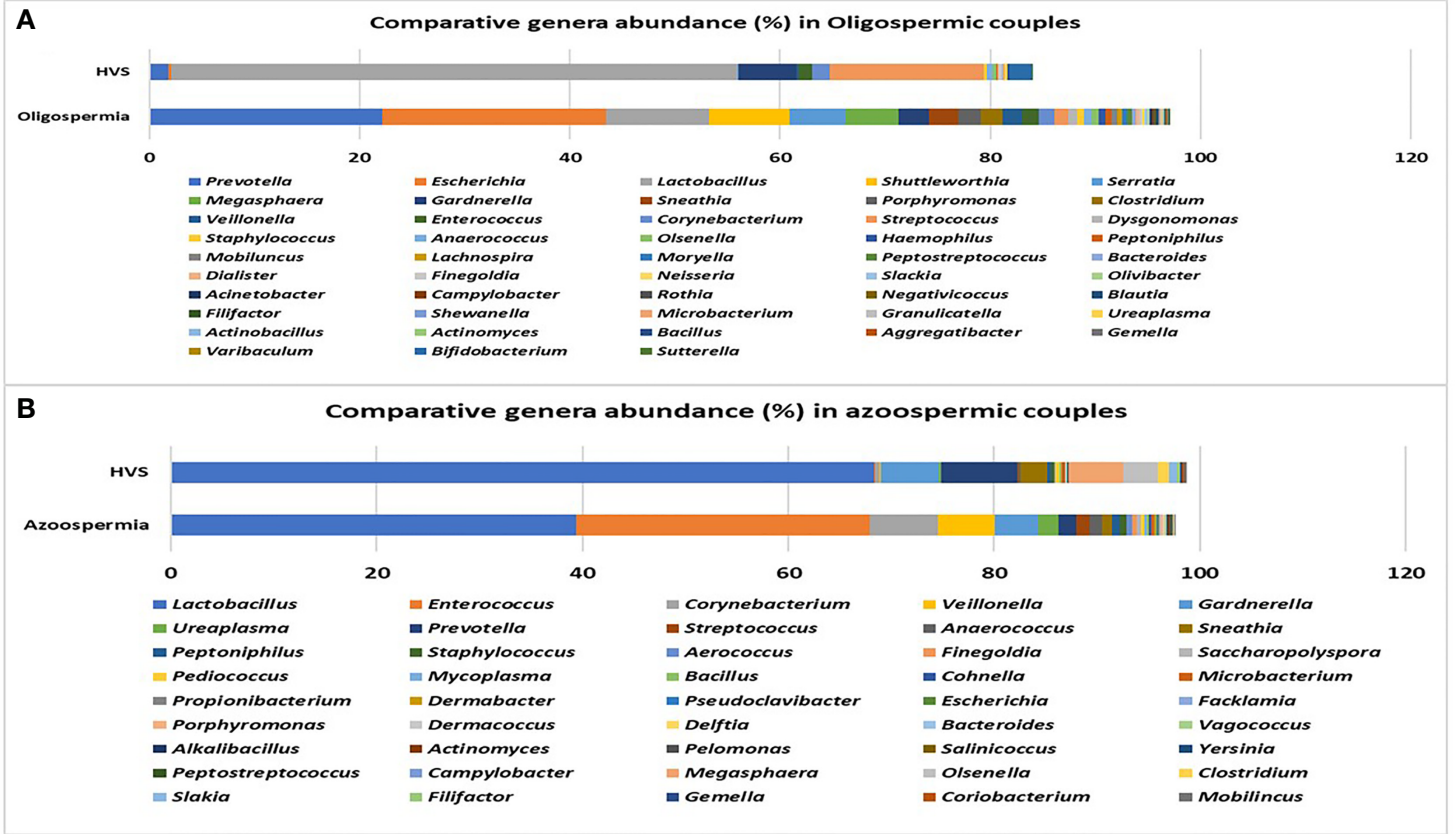
**(A, B)** Comparative relative (%) genera abundance in oligospermia and azoospermia couples.

Among the oligospermic couples, 555 genera were identified in semen samples, while 403 genera were found in the corresponding vaginal samples. *Prevotella* (22.13%) was the most abundant genus in oligospermic semen, followed by *Escherichia* (21.33%), *Lactobacillus* (9.73%), *Shuttleworthia* (7.67%), *Serratia* (5.33%), *Megasphaera* (5.04%), *Gardnerella* (2.85%), *Sneathia* (2.79%), *Porphyromonas* (2.22%), and others. The corresponding vaginal samples had *Lactobacillus* (53.90%) as the most abundant genus, followed by *Streptococcus* (14.68%), *Gardnerella* (5.48%), and others as shown in **Figure 5A**.

### Microbiota Compositions From Azoospermia Couples

The results from azoospermic couples show that 342 genera colonized the semen samples, while 309 genera were found in the corresponding vaginal samples. *Lactobacillus* (39.38%) was the most abundant genus in azoospermic men, followed by *Enterococcus* (28.52%), *Corynebacterium* (6.58%), *Veillonella* (5.53%), *Gardnerella* (4.25%), *Ureaplasma* (1.91%), *Prevotella* (1.82%), and others. The corresponding vaginal samples were colonized mostly by *Lactobacillus* (68.38%), *Prevotella* (7.37%), *Gardnerella* (5.69%), *Megasphaera* (5.23%), *Olsenella* (3.41%), *Sneathia* (2.69%), and others represented in **Figure 5B**.

### Microbiota Compositions From Semen Samples With Leukocytes

We compared the microbiota compositions of nine semen samples with leukocytes (2+ to 3++) and nine semen samples without the presence of leukocytes. Semen samples with leukocytes tend to be significantly less colonized by *Lactobacillus reuteri* group, *Faecalibacterium*, and more inhabited by *Bacteroides* and *Prevotella* (**Supplementary Figure S6**). The corresponding microbiota at genera level from vaginal samples is presented in **Supplementary Figure S7** showing *Lactobacillus* and *Gardnerella* as the most relative abundance. At the species taxonomic level, semen samples with leukocytes had more relative abundance of *Lactobacillus iners* and *Enterococcus faecium* compared with semen without leukocytes as shown in **Supplementary Figure S8**. The corresponding female partners had *Lactobacillus iners* as the most abundant species as shown in **Supplementary Figure S9**. Bacterial metabolic functional genes that were downregulated in the semen samples with leukocytes include but not limited to hemoglobin/transferrin/lactoferrin receptor protein, MFS transporter, OPA family, sugar phosphate sensor protein UhpC as presented in **Supplementary Table S1**.

### Microbiota Compositions in Couples With Positive and Negative IVF Clinical Outcome

This study compared the relative abundance of the microbiota in 12 couples with positive IVF clinical outcome and 24 couples with unsuccessful or negative IVF clinical outcome as shown in **Tables 1A**, **1B**.

**Figure 6:**
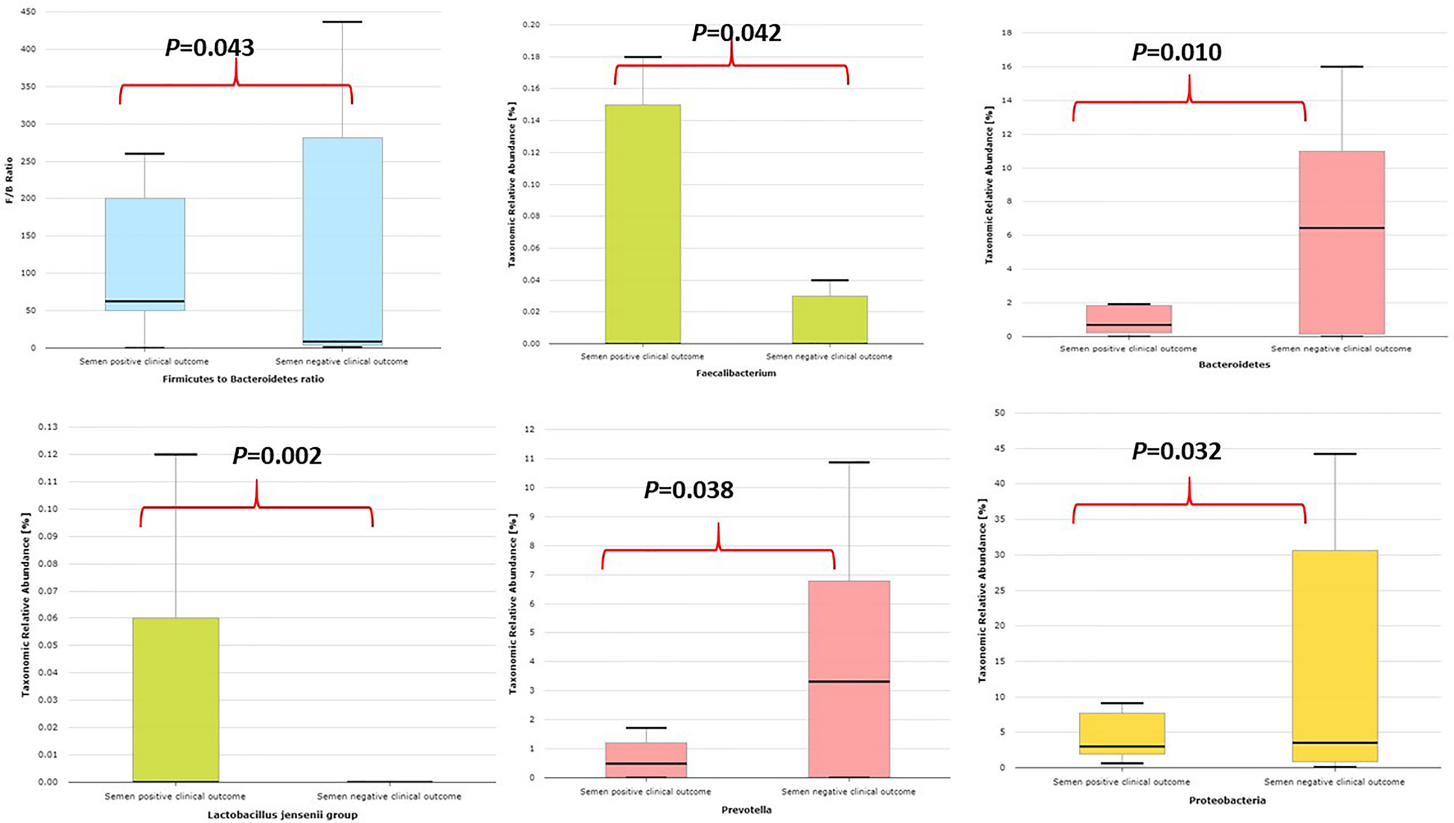
Comparative relative abundance of some selected taxa showing significant difference between semen samples with positive IVF clinical outcome and samples with negative IVF clinical outcome.

Semen samples with positive IVF clinical outcome have less alpha diversity as typified by Shannon index and phylogenetic diversity (**Supplementary Figure S10**) and are significantly colonized by *Lactobacillus jensenii* group and *Faecalibacterium* and significantly less colonized by *Proteobacteria* taxa, *Prevotella*, *Bacteroidetes* taxa, and *Bacteroides* and lower *Firmicutes*/*Bacteroidetes* ratio compared with semen samples with negative IVF clinical outcome as shown in **Figure 6**.

**Figure 7:**
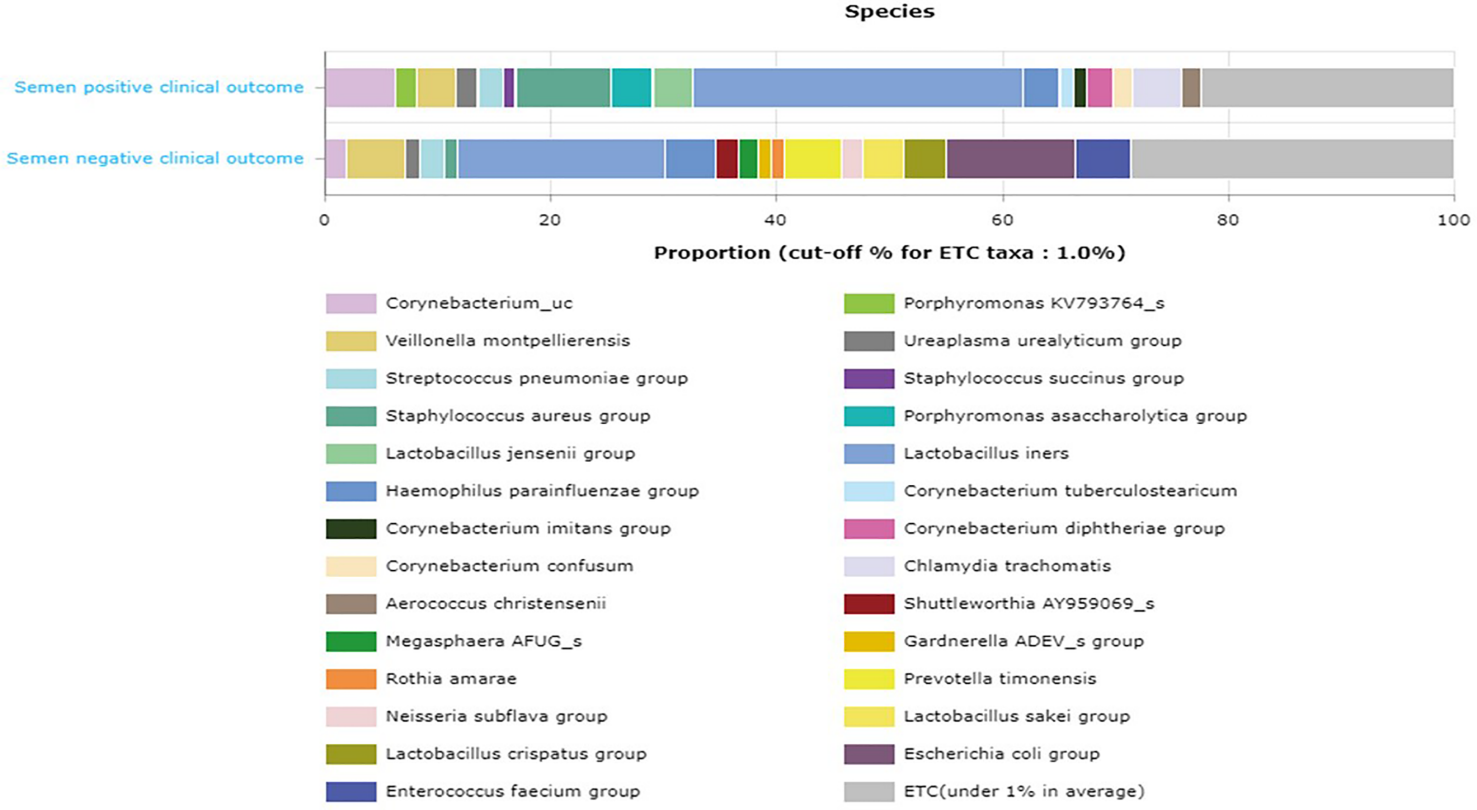
Comparative proportion (cutoff 1.0%) of the relative abundance at the species taxonomic level between semen samples with positive IVF clinical outcome and samples with negative IVF clinical outcome.

The comparative proportion of the relative abundance at the species taxonomic level is represented in **Figure 7**.

LefSe comparison of the taxonomic microbiota biomarkers in the seminal fluid of men that had positive IVF clinical outcome and seminal fluid of those with negative IVF clinical outcome is presented in **Table 2**.

**Table 2:** LefSe comparison of the taxonomic biomarkers in the seminal fluid of men that had positive IVF clinical outcome and seminal fluid of those with negative IVF clinical outcome.

Taxon name	LDA effect size	p-value	p-value (FDR)	Semen positive IVF clinical outcome	Semen negative IVF clinical outcome
*Cutibacterium*	3.50568	0.00034	0.00034	0.76044	0.12494
*Cutibacterium acnes* group	3.45684	0.00067	0.00067	0.66262	0.09221
*Propionibacteriales*	3.48056	0.00068	0.00068	0.82203	0.23874
*Propionibacteriaceae*	3.49112	0.00068	0.00068	0.80718	0.21312
*Salinifilum*	3.70772	0.01579	0.01584	1.02006	0.00000
*Leptotrichiaceae*	3.12386	0.02997	0.03009	0.00000	0.26525
*Facklamia hominis* group	2.60266	0.02997	0.03011	0.00000	0.07949
*Lactobacillus*_uc	2.72540	0.03372	0.03390	0.01318	0.11892
*Actinobacteria*_c	4.76455	0.03899	0.03923	22.04688	10.41718
*Micrococcales*	2.99379	0.04751	0.04783	3.00235	2.80542
*Streptomycetales*	2.92289	0.04825	0.04861	0.16874	0.00170
*Streptomycetaceae*	2.92289	0.04825	0.04865	0.16874	0.00170
*Streptomyces*	2.92289	0.04825	0.04868	0.16874	0.00170
*Cutibacterium avidum*	2.44565	0.04825	0.04871	0.05682	0.00568
*Saccharibacteria*_TM7	2.20252	0.04825	0.04874	0.00000	0.03151
*Saccharimonas*_c	2.20252	0.04825	0.04878	0.00000	0.03151
*Saccharimonas*_o	2.20252	0.04825	0.04881	0.00000	0.03151
*Saccharimonas*_f	2.20252	0.04825	0.04885	0.00000	0.03151
*Prevotella*_uc	2.60734	0.04825	0.04888	0.00000	0.08053
*Actinomyces europaeus* group	2.45905	0.04825	0.04891	0.00000	0.05622
*Streptococcus*_uc	2.02046	0.04825	0.04895	0.00000	0.01467

FDR, False Discovery Rate; LDA, Linear Discriminant Analysis.

In addition, the LefSe comparison of the taxonomic microbiota biomarkers for positive IVF clinical outcome for the couples is presented in **Supplementary Table S2**.

**Figure 8:**
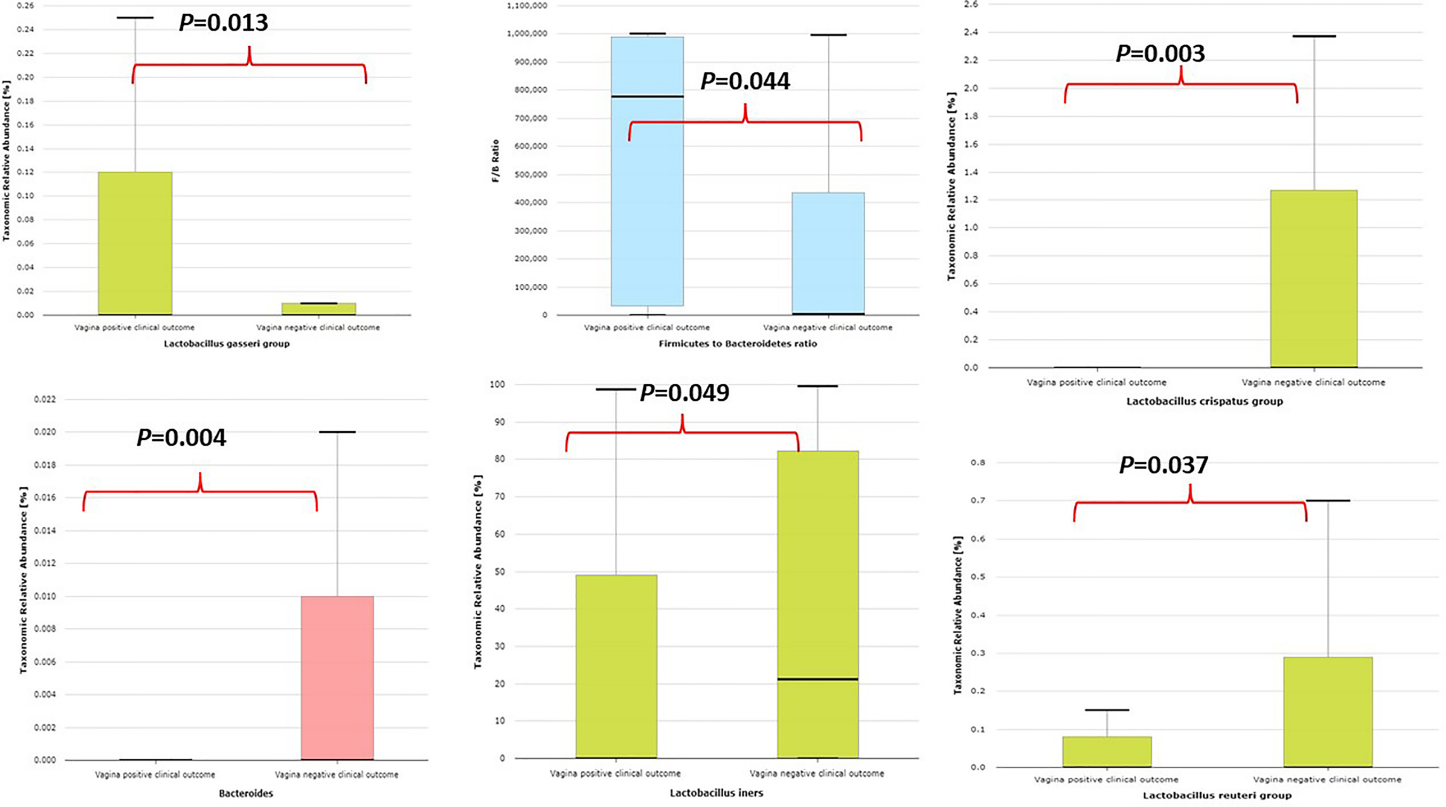
Comparative relative abundance of some selected taxa showing significant difference between vaginal samples with positive IVF clinical outcome and vaginal samples with negative IVF clinical outcome.

Vaginal samples with positive IVF clinical outcome are significantly colonized by *Lactobacillus gasseri* group, higher *Firmicutes/Bacteroidetes* ratio, and significantly less colonized by *Bacteroides*, *Bacteroidetes* taxa, *Lactobacillus reuteri* group, *Lactobacillus iners*, and *Lactobacillus crispatus* as represented in **Figure 8**.

LefSe comparison of the taxonomic microbiota biomarkers in the vaginal samples of women that had positive IVF clinical outcome and vaginal samples of those with negative IVF clinical outcome is presented in **Table 3**.

**Table 3:** LefSe comparison of the taxonomic biomarkers in the vaginal swabs of women that had positive IVF clinical outcome and vaginal swabs of women with negative IVF clinical outcome.

Taxon name	LDA effect size	*p*-value	*p*-value (FDR)	Vagina positive IVF clinical outcome	Vagina negative IVF clinical outcome
*Bacteroidetes*	3.95843	0.00783	0.00783	0.01044	1.82702
*Bacteroidia*	3.95832	0.00889	0.00890	0.00978	1.82624
*Bacteroidales*	3.95832	0.00889	0.00891	0.00978	1.82624
*Prevotellaceae*	3.95382	0.01060	0.01064	0.00267	1.80030
*Prevotella*	3.95366	0.01060	0.01066	0.00267	1.79968
*Citrobacter*	2.17062	0.01579	0.01589	0.02942	0.00000
*Citrobacter koseri*	2.17062	0.01579	0.01591	0.02942	0.00000
***Cutibacterium acnes* group**	3.04732	0.01822	0.01839	0.00000	0.22235
*Enterobacterales*_uc	2.34217	0.01864	0.01884	0.04385	0.00008
*Prevotella timonensis*	3.79064	0.01866	0.01888	0.00224	1.23701
*Cutibacterium*	3.06412	0.02560	0.02594	0.00038	0.23154
*Streptococcus agalactiae*	4.38488	0.03698	0.03767	8.66950	3.81779
*Veillonella*	2.28915	0.04785	0.04881	0.02708	0.06579
***Fusobacteria***	2.40327	0.04825	0.04928	0.00000	0.05037
***Fusobacteria*_c**	2.40327	0.04825	0.04935	0.00000	0.05037
***Fusobacteriales***	2.40327	0.04825	0.04942	0.00000	0.05037
***Mobiluncus***	2.52442	0.04825	0.04962	0.00000	0.06573
***Mobiluncus curtisii* group**	2.51681	0.04825	0.04968	0.00000	0.06468
***Veillonella dispar***	2.37510	0.04825	0.04975	0.00000	0.04684

FDR, False Discovery Rate; LDA, Linear Discriminant Analysis.

The results from PICRUSt indicated that some bacterial metabolic functional genes were upregulated in the semen of men who had positive IVF clinical outcome. For example, bacterial metabolic functional gene orthologs for phenylalanyl-tRNA synthetase beta chain was significantly upregulated (*P*=0.0231) with LDA effect size of 2.0847, when compared with bacterial metabolic genes from seminal fluid of men with negative IVF clinical outcome. Other several bacterial metabolic functional gene orthologs that were significantly upregulated include but not limited to methionyl aminopeptidase (P=0.0231), peptide/nickel transport system permease protein (P=0.0132), chaperonin GroEL (P**=**0.0286), glucose-6-phosphate 1-dehydrogenase (P=0.0374), mycothione reductase (P=0.0318), multicomponent Na+:H+ antiporter subunits D, E, G, A, F, and C (**Supplementary Table S3**).

Similarly, bacterial metabolic functional genes in the vagina of women with positive IVF clinical outcome were upregulated. Notably, iron/zinc/manganese/copper transport system permease protein (P=0.0326) and diphosphoinositol-polyphosphate diphosphatase (P=0.0369) had a twofold increase with positive IVF clinical outcome when compared with negative IVF clinical outcome (**Table 4**).

**Table 4:** Bacterial metabolic functional gene orthologs in the vagina of women with positive IVF clinical outcome and women with negative IVF clinical outcome.

Gene Ortholog	Definition	LDA effect size	p-value	p-value (FDR)	Vagina positive IVF clinical outcome	Vagina negative IVF clinical outcome
K21572	starch-binding outer membrane protein, SusD/RagB family	2.053646	0.006891	0.006898	0.000183145	0.022612675
K21573	TonB-dependent starch-binding outer membrane protein SusC	1.836336	0.006891	0.006899	0.000123226	0.013643597
K01277	dipeptidyl-peptidase III	1.106637	0.007833	0.007843	1.51541E-05	0.00237178
K00895	diphosphate-dependent phosphofructokinase	0.934877	0.010441	0.010463	5.83556E-05	0.001579854
K16363	UDP-3-O-[3-hydroxymyristoyl] N-acetylglucosamine deacetylase/3-hydroxyacyl-[acyl-carrier-protein] dehydratase	0.903303	0.024279	0.024405	1.06454E-05	0.001411429
K11705	iron/zinc/manganese/copper transport system permease protein	1.112538	0.032613	0.032839	0.004644898	0.002253295
K07766	diphosphoinositol-polyphosphate diphosphatase	1.112288	0.036982	0.037275	0.004642652	0.002252543

FDR, False Discovery Rate; LDA, Linear Discriminant Analysis.

## Discussion

To our knowledge, this is the first study from Nigeria that utilized next-generation sequencing (NGS) technology to determine the microbiota compositions of the seminal fluids and vaginal swabs from couples seeking assisted reproductive health care. This study showed a higher percentage of primary infertility (69.0%) when compared with subjects who had secondary infertility (31.0%). The high rate of primary infertility in this study was in agreement with the previous results of Ikechebelu et al. (Ikechebelu, 2003; Ikechebelu et al., 2003).

Clinical pregnancy determined with ultrasound scan showed 33% (12/36) positive IVF outcomes, which led to 91.6% (11/12) live delivery, and only 8.3% (1/12) clinical pregnancy loss (miscarriage) occurred. This clinical pregnancy rate (33%) is similar to our previous study (Ikechebelu et al., 2016) and very close to the overall pregnancy rate (36%) as reported by Haahr et al. (2019). Among couples diagnosed with secondary infertility, only 11% (4/36) had positive clinical IVF outcome, compared with 22% (8/36) of couples with primary infertility. The 16S rRNA results, when taken together, revealed that the semen microbiota is highly polymicrobial, typified by alpha diversity indexes such as Shannon index and phylogenetic diversity, but low in species concentrations, as shown in a similar study by Mandar et al. (2015). This finding was consistent with our previous pilot study of seminal fluids in a tertiary hospital that revealed varying bacterial diversities that are unique in each sample in contrast to culture-dependent methods (Ndiokwere et al., 2019). The origin of these diverse bacteria in seminal fluids is still not fully determined, but interestingly, most of the bacterial species are closely related to human vaginal microbes (Ravel et al., 2010; Anukam et al., 2019; Okoli et al., 2019), urine microbiota (Nelson et al., 2010), and urethra (Riemersma et al., 2003). In contrast, the vaginal microbiota had higher species concentrations with less bacterial diversity. The family taxa *Lactobacillaceae* and the *Lactobacillus* genus were significantly higher in the vagina than in the semen (61.74 *vs* 43.86%), which is consistent with several studies showing that the healthy vagina is colonized by *Lactobacilli* that help to fend off pathogenic microbes by increasing the pH and preventing urogenital infections (Amabebe and Anumba, 2018). However, previous studies have shown that *Lactobacillus* is part of a normal microbiota of the seminal fluid in healthy subjects (Ivanov et al., 2009 **)**. It is noteworthy that couples shared many of the predominant genera (56%) and some species (41%) in their reproductive tracts, such as *Gardnerella vaginalis*, *Lactobacillus iners*, *Lactobacillus japonicus*, *Lactobacillus jensenii*, and *Lactobacillus agilis*, which suggests that a healthy vagina or vagina with dysbiosis could influence the reproductive tract microbiota composition of the sexual partner, and *vice versa*. Similar observations have been documented on the skin microbiome of cohabiting couples (Ross et al., 2017). Another interesting finding showed that couples have the same genera but different species, though this is not surprising as the physiological condition of the vagina is acidic while semen is alkaline. This study shows that *Gardnerella* (25.45 *vs* 5.86%) and *Veillonella* (7.78 *vs* 0.04%) were more in abundance in the seminal fluids with normospermia than the corresponding vagina microbiota, although *Gardnerella vaginalis* has severally been associated with bacterial vaginosis (Anukam et al., 2019). The physiological role of *Gardnerella vaginalis* in normozoospermic healthy subjects is yet to be determined as Weng et al. (2014) found that *Lactobacillus*, *Gardnerella*, *Propionibacterium*, and *Atopobium* were relatively more in abundance and significantly present in the normal semen samples. In this study, we found that relative signatures of bacterial communities could be used to disentangle semen categories. Men seeking reproductive health care in the tested population, though found to be normozoospermic, tend to have more *Lactobacillus* >*Gardnerella* >*Veillonella* >*Corynebacterium*, while the female partners have more *Lactobacillus* >*Prevotella* >*Gardnerella* >*Streptococcus*. This suggests that the source of their infertility could probably be more than altered bacterial communities. A similar finding was reported by Hou *et al. (* Hou et al., 2013) showing that infertile subjects did not have altered or unusual semen bacterial communities compared to normal sperm donors. The men categorized as oligospermia tend to have more *Prevotella* >*Escherichia* > *Lactobacillus* >*Shuttleworthia* >*Serratia* >*Megasphaera* >*Gardnerella* >*Sneathia*, and their female partners tend to have more *Lactobacillus* >*Streptococcus* >*Gardnerella* >*Lactococcus* >*Bifidobacterium* >*Prevotella*. It appears that oligospermia men may be under the influence of these pathogens that overwhelm *Lactobacillus*’s protective activities. The factors responsible for these microbial community differences observed in oligospermic couples are yet to be delineated. In contrast, azoospermia men in these cohorts were observed to have more *Lactobacillus* >*Enterococcus* >*Corynebacterium* >*Veillonella* >*Gardnerella* >*Ureaplasma* >*Prevotella*, while their female partners have more *Lactobacillus* >*Prevotella* >*Gardnerella* >*Megasphaera* >*Olsenella* >*Sneathia* >*Peptoniphillus*, and other BV-associated bacteria. Previous studies have reported that several bacteria, including *Lactobacillus iners*, *Gardnerella vaginalis*, *Escherichia faecalis*, *E. coli*, and *Staphylococcus aureus*, are associated with male infertility (Franasiak and Scott, 2015; Javurek et al., 2016). These bacterial taxa are common to both semen and vagina. The caveat is that they occur at different proportions in the semen and vaginal niche. The differences in these bacterial communities in these partners may be due to sexual intercourse, episodes of receptive oral sex, and anal sex before vaginal intercourse, which has been reported to influence vaginal and genital tract microbiota in infertile couples (Beigi et al., 2005). The presence of leukocytospermia or pyospermia may have been triggered by the presence of *Gardnerella*, *Prevotella*, and other BV-associated bacteria, which leads to alteration in the partner’s vaginal microbiota, and BV has been associated with a 40% increase in the risk of preterm birth (Hillier et al., 1995) and infertility (Al-Awadhi et al., 2013). The occurrence of BV-related microbiota in semen suggests a possible reservoir and supports the concept of sexual transmission of BV, besides semen’s alkaline properties, which may alter the acidic pH of the vagina, leading to BV (Gallo et al., 2011). An earlier study examined adherence, biofilm formation, and cytotoxicity *in vitro* for *G. vaginalis* strains isolated from women with BV as well as other BV-associated bacteria, including *Atopobium*, *Prevotella*, and *Mobiluncus* (Patterson et al., 2010). In terms of the impact of BV on IVF, several authors have observed high rates of BV on women with reproductive IVF failure and adverse pregnancy outcome (Spandorfer et al., 2001; Wilson et al., 2002; Wittemer et al., 2004), which corroborates with the findings in this study. Surprisingly, in this study, *Lactobacillus crispatus* was found to be less colonized in women with positive IVF clinical pregnancy outcome in contrast to the work of Haahr et al. (2019). Instead, *Lactobacillus gasseri* was significantly associated with positive IVF clinical outcome. The negative IVF clinical outcome observed in these cohorts of men and women may have been due to the induction of inflammatory response that inhibited the sperms from fertilizing the ovum. Although, Vergaro et al. (2019) showed that vaginal microbiota profile at the time of embryo transfer does not affect the live birth rate in IVF cycles, however, the study has been challenged as it was ladened with poor diagnosis and flawed conclusions (Haahr et al., 2019). The microbiota biomarkers identified in women with negative IVF clinical outcomes in this study point towards an infectious perturbation of the IVF process, which corroborates the findings of Haahr et al. (2016). For example, in this study, the significant increase of *Bacteroidales*, *Prevotellaceae*, *Mobiluncus curtisii*, and *Cutibacterium acnes* in the vagina of those with negative IVF clinical outcome lends credence to previous observations on causes of IVF failure (Onemu and Ibeh, 2001; Al-Awadhi et al., 2013). *Cutibacterium acnes* are involved in the inflammation of the skin by secreting lipase enzymes, which metabolize sebum into free fatty acids, but its activity in the vagina of women with negative IVF clinical outcomes is yet to be determined. A recent study has demonstrated that IVF does not occur in a sterile environment, and the presence of *Staphylococcus* sp. and *Alphaproteobacteria* is associated with clinical indicators such as sperm and embryo quality (Štšepetova et al., 2020).

Transferrin/lactoferrin receptor proteins were significantly upregulated in the bacterial metabolic genes found in semen samples without leukocytes and with positive IVF clinical outcomes when compared with those that had negative IVF clinical outcomes. It should be noted that lactoferrin is a member of the iron-binding transferrin proteins known to have antimicrobial properties and plays a significant role in the mucosal immune response. In addition, lactoferrin aids neutrophils by regulating hydroxyl radical production and participates in the secretion of IgA antibodies (Spik and Montreuil, 1983). It is very interesting to observe that some bacterial metabolic functional genes were upregulated in the vagina of women with positive IVF outcomes. For example, metal ions transport system permease protein such as iron/zinc/manganese/copper were upregulated. These permease systems are required in many biological processes as components of metalloproteins and serve as cofactors or structural elements for enzymes. It should be noted that some bacteria employ a variety of metal uptake and export mechanisms to regulate metal homeostasis by numerous transcriptional regulators (Porcheron et al., 2013).

## Conclusions

The fact that semen samples of men with positive IVF clinical outcomes were significantly colonized by *Lactobacillus jensenii* group and *Faecalibacterium* and significantly less colonized by *Proteobacteria* taxa, *Prevotella*, *Bacteroidetes* taxa suggests an association between semen and vaginal microbiota correlation. *Lactobacillus gasseri* was significantly associated with positive IVF clinical outcome in women. In addition, this study has opened a window of the possibility of using clinically tested probiotics therapy for men and women before IVF treatment. The need for implementing this approach has been advocated (Verstraelen and Senok, 2005; Garcia-Velasco et al., 2017).

## Data Availability Statement

The datasets presented in this study can be found in online repositories. The names of the repository/repositories and accession number(s) can be found below: the 72 raw sequence reads (FASTQ files) have been deposited on the Sequence Read Archive (SRA) of the National Center for Biotechnology Information (NCBI) with Project Accession Number PRJNA762524 (https://www.ncbi.nlm.nih.gov/bioproject/?term=PRJNA762524).

## Ethics Statement

The studies involving human participants were reviewed and approved by the Ethics Review Committee on Human Research from Nnamdi Azikiwe University Teaching Hospital (Ref. # NAUTH/CS/66/VOL11/175/2018/111). The patients/participants provided their written informed consent to participate in this study.

## Author Contributions

KCA designed the study and sourced for funding; SIO and KCA were responsible for taxonomic data organization, initial analysis and manuscript drafting; KCA was responsible for bioinformatic analysis; JII and SIO were involved in clinical evaluation and the collection of samples; KCA, JII and NRA were the principal investigators and participated in the final design of the study, coordination and drafting the manuscript. All authors contributed to the interpretation of data and made a substantial, direct, and intellectual contribution to the work and approved it for publication.

## Funding

This study was partly funded by uBiome Inc. San Francisco, USA, under an academic consortium grant-in-kind award to KA and Uzobiogene Genomics, London, Ontario, Canada. However, uBiome did not participate in the design of the study, collection, analysis, interpretation of data and in writing the manuscript.

## Conflict of Interest

The authors declare that the research was conducted in the absence of any commercial or financial relationships that could be construed as a potential conflict of interest.

## Publisher’s Note

All claims expressed in this article are solely those of the authors and do not necessarily represent those of their affiliated organizations, or those of the publisher, the editors and the reviewers. Any product that may be evaluated in this article, or claim that may be made by its manufacturer, is not guaranteed or endorsed by the publisher.

## Supplementary Material

Supplementary Figure 1 Principal Coordinate Analysis (PCoA) with the Bray-Curtis clustering in bacterial community diversities between semen and vagina.

Supplementary Figure 2 The rarefaction curve showing the number of reads between semen samples and vaginal samples.

Supplementary Figure 3 Venn diagram showing number of taxa exclusive to normospermia, oligospermia, azoospermia, and taxa that were common to the semen categories.

Supplementary Figure 4 Comparative stacked relative (%) abundance of taxonomic phyla between semen and vagina in normospermia.

Supplementary Figure 5 Comparative stacked relative (%) abundance of taxonomic genera between semen and vagina in normospermia.

Supplementary Figure 6 Comparative relative abundance of some selected taxa showing significant difference between semen samples without leukocytes and semen samples leukoocytes.

Supplementary Figure 7 Relative (%) abundance of taxonomic genera in the vaginal samples of the corresponding pyospermia samples.

Supplementary Figure 8 Comparative relative (%) abundance of some species taxa between semen samples with leukocytes and semen without leukocytes.

Supplementary Figure 9 Relative (%) abundance of taxonomic species in the vaginal samples of the corresponding pyospermia samples.

Supplementary Figure 10 Comparative alpha diversities between semen samples with positive IVF clinical outcome and semen samples with negative IVF clinical outcome.
