# Supplementary material for: Microbiome Compositions From Infertile Couples Seeking In Vitro Fertilization, Using 16S rRNA Gene Sequencing Methods: Any Correlation to Clinical Outcomes?
Source: Front Cell Infect Microbiol. 2021 Oct 1;11:709372. doi: 10.3389/fcimb.2021.709372 (PMC8517523; doi:10.3389/fcimb.2021.709372)
Supplement: Supplementary file 2 [file DataSheet_2.pdf]

Table S1:

Gene Ortholog	Metabolic Functional Gene Definition	LDA effect size	p-value	p-value (FDR)	Normospermia without leucocytes	Semen with Leukocytes
K02025	multiple sugar transport system permease protein	2.261725843	0.012724	0.012752175	0.064354389	0.100693286
K00557	tRNA (uracil-5-)-methyltransferase	1.874490605	0.012724	0.012758059	0.015985129	0.001204857
K16087	hemoglobin/transferrin/lactoferrin receptor protein	2.070152298	0.01991	0.020019188	0.025014425	0.001708239
K02119	V/A-type H+/Na+-transporting ATPase subunit C	1.710055455	0.024682	0.024843508	0.002815807	0.012874332
K10806	acyl-CoA thioesterase YciA	1.841834075	0.030413	0.030660244	0.017565963	0.003870828
K07783	MFS transporter, OPA family, sugar phosphate sensor protein UhpC	1.722774099	0.030413	0.030702979	0.011095006	0.000731635
K02122	V/A-type H+/Na+-transporting ATPase subunit F	1.697757071	0.030413	0.030712492	0.00294449	0.012716599
K01995	branched-chain amino acid transport system ATP-binding protein	2.209931713	0.037253	0.037677881	0.09816811	0.130399195
K03808	paraquat-inducible protein A	1.69974082	0.037253	0.037698353	0.010599677	0.000781914
K03576	LysR family transcriptional regulator, regulator for metE and metH	1.87590023	0.037253	0.03770128	0.019489044	0.004660097
K03604	LacI family transcriptional regulator, purine nucleotide synthesis repressor	1.949109778	0.045361	0.046168073	0.027013054	0.009424649
K02806	PTS system, nitrogen regulatory IIA component	1.980305469	0.045361	0.046186102	0.026697121	0.007783834
K03583	exodeoxyribonuclease V gamma subunit	1.886805413	0.045361	0.046240272	0.019449782	0.004238622
K02453	general secretion pathway protein D	1.818376563	0.045361	0.046247505	0.017774012	0.004809466

Table S2:

Taxon name	LDA effect size	p-value	p-value (FDR)	Semen positive IVF clinical outcome	Vagina positive IVF clinical outcome
Firmicutes	5.09401	0.02782	0.03009	56.34075	81.17426
Bacilli	5.21938	0.01975	0.02116	47.3827	80.52727
Lactobacillales	5.33029	0.00949	0.00997	37.4993	80.28656
Lactobacillaceae	5.18136	0.04121	0.04617	32.84479	63.21107
Lactobacillus	5.18089	0.04121	0.04624	32.83665	63.16992
Actinobacteria	4.94624	0.00783	0.00817	22.27609	4.60474
Actinobacteria_c	4.96207	0.00428	0.0044	22.04688	3.71965
Corynebacteriales	4.84637	0.00643	0.00666	14.81763	0.7768
Corynebacteriaceae	4.84196	0.00643	0.00667	14.65991	0.76095
Corynebacterium	4.84186	0.00949	0.00999	14.64746	0.75181
Bacillales	4.68318	0.00783	0.00818	9.8834	0.2407
Staphylococcaceae	4.6722	0.01137	0.012	9.62297	0.22106
Staphylococcus	4.67058	0.00776	0.00809	9.57409	0.20708
Staphylococcus aureus group	4.61706	0.01137	0.01201	8.48793	0.20708
Bacteroidetes	4.58572	0.00134	0.00136	7.71487	0.01044
Bacteroidia	4.58477	0.0017	0.00173	7.69734	0.00978
Bacteroidales	4.58477	0.0017	0.00174	7.69734	0.00978
Proteobacteria	4.29491	0.00949	0.00996	6.98912	10.93298
Corynebacterium_uc	4.47953	0.01549	0.01653	6.16697	0.13377
Porphyromonadaceae	4.44654	0.04929	0.05568	5.59843	0.00656
Porphyromonas	4.44654	0.04929	0.05576	5.59843	0.00656
Gammaproteobacteria	4.43926	0.01369	0.01448	5.31457	10.81351
Negativicutes	4.38691	0.03556	0.03956	5.14508	0.27072
Veillonellales	4.38553	0.04183	0.04699	5.12968	0.27072
Veillonellaceae	4.38553	0.04183	0.04706	5.12968	0.27072

Veillonella	4.35812	0.00142	0.00144	4.58879	0.02708
Pasteurellales	4.21005	0.00685	0.00711	3.24644	0.00264
Pasteurellaceae	4.21005	0.00685	0.00712	3.24644	0.00264
Haemophilus	4.20881	0.00685	0.00713	3.23717	0.00264
Haemophilus parainfluenzae group	4.20555	0.00576	0.00596	3.21037	0
Micrococcales	4.15838	0.00039	0.00039	3.00235	0.12248
Tissierellia	4.04951	0.00181	0.00185	2.58313	0.34196
Tissierellales	4.04951	0.00181	0.00185	2.58313	0.34196
Peptoniphilaceae	4.04951	0.00181	0.00185	2.58313	0.34196
Corynebacterium diphtheriae group	4.03548	0.02629	0.02836	2.29907	0.12913
Prevotellaceae	4.00565	0.01103	0.01162	2.02863	0.00267
Prevotella	4.00166	0.03293	0.03659	2.01009	0.00267
KV793764_s	3.97497	0.03181	0.03497	1.8878	0
Corynebacterium confusum	3.94526	0.04901	0.05529	1.79477	0.03182
Micrococcaceae	3.77796	0.00124	0.00126	1.24546	0.0462
Pseudomonadales	3.72559	0.00086	0.00087	1.07523	0.0123
Staphylococcus succinus group	3.72754	0.01393	0.01481	1.06775	0
Clostridia	3.67147	0.03701	0.04123	0.97261	0.03431
Clostridiales	3.67147	0.03701	0.04129	0.97261	0.03431
Betaproteobacteria	3.61248	0.00027	0.00027	0.92971	0.11096
Moraxellaceae	3.64364	0.00086	0.00087	0.89077	0.01068
Propionibacteriales	3.61278	0.00005	0.00005	0.82203	0.00235
Propionibacteriaceae	3.60485	0.00005	0.00005	0.80718	0.00235
Acinetobacter	3.58208	0.00328	0.00336	0.77442	0.01068
Cutibacterium	3.57999	0.00004	0.00004	0.76044	0.00038
Burkholderiales	3.49195	0.00035	0.00035	0.72768	0.10832
Finegoldia	3.48308	0.00112	0.00113	0.71095	0.10429
Brevibacteriaceae	3.51418	0.02031	0.02184	0.69968	0.04665
Brevibacterium	3.51418	0.02031	0.02187	0.69968	0.04665
Prevotella timonensis	3.52223	0.04039	0.04519	0.66734	0.00224

Cutibacterium acnes group	3.52039	0.00002	0.00002	0.66262	0
Finegoldia magna	3.4283	0.00088	0.00089	0.63434	0.10011
Comamonadaceae	3.42454	0.00344	0.00353	0.5877	0.05745
Rothia	3.46403	0.03293	0.03654	0.58267	0.00076
Dermabacteraceae	3.38255	0.00516	0.00531	0.49778	0.0156
Veillonella ratti group	3.34061	0.03181	0.03492	0.4378	0
Delftia	3.25927	0.0194	0.02073	0.39414	0.03163
Delftia tsuruhatensis group	3.25927	0.0194	0.02076	0.39414	0.03163
Microbacteriaceae	3.2494	0.00588	0.00608	0.36765	0.01404
Micrococcus luteus group	3.23728	0.04039	0.04513	0.34509	0.00016
Acinetobacter haemolyticus group	3.05244	0.03181	0.03464	0.22508	0
Actinomycetales	2.98079	0.00878	0.00919	0.19705	0.01071
Actinomycetaceae	2.98079	0.00878	0.00921	0.19705	0.01071
Zimmermannella	2.95452	0.03181	0.03459	0.17955	0
Zimmermannella bifida	2.95452	0.03181	0.03487	0.17955	0
Veillonella dispar	2.93584	0.01393	0.01483	0.17069	0
Streptomycetales	2.92707	0.03181	0.03445	0.16874	0
Streptomycetaceae	2.92707	0.03181	0.0345	0.16874	0
Streptomyces	2.92707	0.03181	0.03454	0.16874	0
Yersiniaceae	2.72263	0.02734	0.02953	0.1372	0.03354
Intrasporangiaceae	2.8171	0.01393	0.01475	0.12868	0
Brachybacterium	2.81245	0.00543	0.00559	0.12776	0.00153
Brachybacterium faecium group	2.81385	0.00543	0.0056	0.12776	0.00131
Actinomyces	2.8047	0.04552	0.05129	0.12566	0.00961
Enhydrobacter	2.76726	0.01393	0.01477	0.11636	0
Enhydrobacter aerosaccus group	2.76726	0.01393	0.01479	0.11636	0
Yersinia	2.7521	0.01978	0.02122	0.11307	0.00568
Yersinia pestis group	2.7521	0.01978	0.02124	0.11307	0.00568
Pseudomonas stutzeri group	2.63966	0.03181	0.03478	0.08686	0
Mobiluncus	2.55388	0.03181	0.03506	0.07139	0

Mobiluncus curtisii group	2.55388	0.03181	0.03511	0.07139	0
Acinetobacter junii group	2.56823	0.03181	0.03501	0.06841	0
Brevibacterium iodinum group	2.43642	0.03181	0.03468	0.05228	0
Cutibacterium granulosum	2.32763	0.03181	0.03473	0.041	0
Ralstonia syzygii group	2.56562	0.03181	0.03482	0.01404	0
Lactobacillus_uc	3.46664	0.00174	0.00178	0.01318	0.59829
Streptococcus agalactiae	4.63698	0.01393	0.01485	0	8.6695
Anaerococcus vaginalis group	2.12563	0.03181	0.0352	0	0.02547

Table S3:

Gene Ortholog	Definition	LDA effect size	p-value	p-value (FDR)	Semen positive IVF clinical outcome	Semen negative IVF clinical outcome
K01972	DNA ligase (NAD+)	2.127514	0.025784	0.027006	0.14999749	0.123372278
K03111	single-strand DNA-binding protein	1.989716	0.047509	0.051646	0.11392864	0.094596717
K02342	DNA polymerase III subunit epsilon	2.237197	0.020776	0.021533	0.113473107	0.079140673
K03466	DNA segregation ATPase FtsK/SpoIIIE, S-DNA-T family	2.047748	0.013243	0.013504	0.107140416	0.085016167
K01890	phenylalanyl-tRNA synthetase beta chain	2.084727	0.023163	0.02414	0.100749475	0.076641049
K02335	DNA polymerase I	2.108086	0.002953	0.002964	0.10072175	0.075270088
K01265	methionyl aminopeptidase	1.987601	0.023163	0.024148	0.10020196	0.080964916
K01462	peptide deformylase	1.990179	0.043071	0.046367	0.09983316	0.080480433
K07042	probable rRNA maturation factor	2.015016	0.038989	0.041653	0.099490781	0.078987216
K02033	peptide/nickel transport system permease protein	2.129036	0.013243	0.013497	0.098642617	0.071923204
K00655	1-acyl-sn-glycerol-3-phosphate acyltransferase	2.028256	0.038989	0.041735	0.095686398	0.074541928
K03070	preprotein translocase subunit SecA	1.942646	0.020776	0.021523	0.094463108	0.077137421
K03177	tRNA pseudouridine55 synthase	2.079707	0.043071	0.046331	0.092473783	0.068644748
K04077	chaperonin GroEL	2.139938	0.028658	0.030169	0.089787416	0.062383681
K07058	membrane protein	2.047924	0.014856	0.015206	0.082515965	0.060382606
K03665	GTPase	1.899305	0.03524	0.037399	0.082092403	0.066431247
K01772	protoporphyrin/coproporphyrin ferrochelatase	2.052958	0.020776	0.021517	0.074863913	0.052470186
K03327	multidrug resistance protein, MATE family	2.203422	0.047509	0.051642	0.073717078	0.105465668
K01239	purine nucleosidase	2.108919	0.043071	0.046305	0.073311234	0.047810338

K02483	two-component system, OmpR family, response regulator	2.047107	0.03524	0.037454	0.07263009	0.050538717
K00626	acetyl-CoA C-acetyltransferase	1.99962	0.03524	0.037419	0.069818706	0.05003624
K00036	glucose-6-phosphate 1-dehydrogenase	1.975893	0.03524	0.037469	0.066485462	0.047765376
K09167	uncharacterized protein	2.046138	0.038989	0.041791	0.045411383	0.023369705
K02021	putative ABC transport system ATP-binding protein	1.958238	0.023163	0.024076	0.041531088	0.023564754
K06919	putative DNA primase/helicase	2.013319	0.028658	0.03016	0.037171357	0.057594173
K00656	formate C-acetyltransferase	2.070174	0.014856	0.015198	0.032817127	0.056124477
K02022	HlyD family secretion protein	1.827474	0.025784	0.026946	0.032391741	0.019148527
K00847	fructokinase	1.932501	0.011787	0.011997	0.031899598	0.048820644
K07665	two-component system, OmpR family, copper resistance phosphate regulon response regulator CusR	1.979416	0.025784	0.026969	0.030221569	0.049095685
K07636	two-component system, OmpR family, phosphate regulon sensor histidine kinase PhoR	1.949764	0.047509	0.051486	0.030145419	0.047760752
K18851	diacylglycerol O-acyltransferase / trehalose O-mycolyltransferase	1.973155	0.049845	0.054394	0.028820919	0.010219762
K00128	aldehyde dehydrogenase (NAD+)	1.915927	0.043071	0.046312	0.028683223	0.012403259
K04069	pyruvate formate lyase activating enzyme	2.149586	0.006432	0.006486	0.027926333	0.055950172
K05568	multicomponent Na+:H+ antiporter subunit D	1.927471	0.043071	0.04632	0.024558915	0.007835
K05569	multicomponent Na+:H+ antiporter subunit E	1.921343	0.038989	0.04163	0.024144598	0.00765781
K05571	multicomponent Na+:H+ antiporter subunit G	1.910777	0.038989	0.041709	0.023648463	0.007562758
K05565	multicomponent Na+:H+ antiporter subunit A	1.916768	0.043071	0.046422	0.023639464	0.007327532
K05570	multicomponent Na+:H+ antiporter subunit F	1.901026	0.038989	0.041657	0.023096116	0.007371991

K00516	lytic starch monooxygenase	1.907872	0.016639	0.017078	0.022382885	0.006405736
K07741	anti-repressor protein	1.996787	0.016639	0.017098	0.021925715	0.04157828
K05567	multicomponent Na+:H+ antiporter subunit C	1.852899	0.03524	0.037498	0.021176543	0.007122792
K17883	mycothione reductase	1.837484	0.031803	0.033602	0.020921679	0.007364984
K07007	3-dehydro-bile acid Delta4,6-reductase	1.884045	0.031803	0.033694	0.020019634	0.035133099
K06871	uncharacterized protein	2.007904	0.020776	0.021548	0.019971188	0.040138492
K14059	integrase	2.082284	0.043071	0.046316	0.019184477	0.043156532
K07175	PhoH-like ATPase	1.854199	0.016639	0.017104	0.019145187	0.00504872
K04758	ferrous iron transport protein A	1.861288	0.028658	0.03012	0.01836619	0.032697865
K03737	pyruvate-ferredoxin/flavodoxin oxidoreductase	1.871032	0.023163	0.024091	0.018221391	0.032882862
K11904	type VI secretion system secreted protein VgrG	2.217267	0.013243	0.013514	0.017533436	0.050316935
K07089	uncharacterized protein	1.82063	0.020776	0.021518	0.013720232	0.026753276
K19337	RpiR family transcriptional regulator, carbohydrate utilization regulator	2.090217	0.028658	0.030141	0.013489406	0.037907035
K12678	autotransporter family porin	1.967311	0.014856	0.015204	0.013421248	0.031771107
K07484	transposase	2.53569	0.031803	0.033681	0.010953154	0.079415756
K09812	cell division transport system ATP-binding protein	1.848876	0.011787	0.011989	0.009325629	0.023247895
K08978	bacterial/archaeal transporter family protein	1.899764	0.008234	0.008326	0.009106418	0.024784274
K00651	homoserine O-succinyltransferase/O-acetyltransferase	1.881026	0.002953	0.002963	0.008456631	0.023464036
K20861	FMN hydrolase / 5-amino-6-(5-phospho-D-ribitylamino)uracil phosphatase	2.063187	0.011787	0.011993	0.007491599	0.030423796
K04027	ethanolamine utilization protein EutM	1.81211	0.002579	0.002588	0.002144374	0.014920324
